# Involvement of mTOR signaling in sphingosylphosphorylcholine-induced hypopigmentation effects

**DOI:** 10.1186/1423-0127-18-55

**Published:** 2011-08-13

**Authors:** Hyo-Soon Jeong, Seung Hoon Lee, Hye-Young Yun, Kwang Jin Baek, Nyoun Soo Kwon, Kyoung-Chan Park, Dong-Seok Kim

**Affiliations:** 1 Department of Biochemistry, Chung-Ang University College of Medicine, 221 Heukseok-dong Dongjak-gu, Seoul 156-756, Republic of Korea; 2 Department of Dermatology, Seoul National University Bundang Hospital, 300 Gumi-dong, Bundang-gu, Seongnam-si, Kyoungki-do 463-707, Republic of Korea

**Keywords:** Akt/LC3 II/Melanocytes/mTOR/sphingosylphosphorylcholine

## Abstract

**Background:**

Sphingosylphosphorylcholine (SPC) acts as a potent lipid mediator and signaling molecule in various cell types. In the present study, we investigated the effects of SPC on melanogenesis and SPC-modulated signaling pathways related to melanin synthesis.

**Methods:**

Melanin production was measured in Mel-Ab cells. A luciferase assay was used to detect transcriptional activity of the MITF promoter. Western blot analysis was performed to examine SPC-induced signaling pathways.

**Results:**

SPC produced significant hypopigmentation effects in a dose-dependent manner. It was found that SPC induced not only activation of Akt but also stimulation of mTOR, a downstream mediator of the Akt signaling pathway. Moreover, SPC decreased the levels of LC3 II, which is known to be regulated by mTOR. Treatment with the mTOR inhibitor rapamycin eliminated decreases in melanin and LC3 II levels by SPC. Furthermore, we found that the Akt inhibitor LY294002 restored SPC-mediated downregulation of LC3 II and inhibited the activation of mTOR by SPC.

**Conclusions:**

Our data suggest that the mTOR signaling pathway is involved in SPC-modulated melanin synthesis.

## Background

Melanin, a pigment found in hair, eyes, and skin, is produced by melanocytes and its synthesis is promoted by various stimulators such as UV irradiation, hormones, and cytokines [1-3]. At least 3 enzymes are required for melanin synthesis. Tyrosinase catalyses the first 2 rate-limiting steps of melanogenesis, whereas tyrosinase-related protein 1 (TRP1) and TRP2 convert melanin into different types. Microphthalmia-associated transcription factor (MITF) is a critical factor in melanin synthesis because it modulates the expression of tyrosinase, TRP1, and TRP2 [4,5]. Thus, much attention has been directed toward finding materials that regulate the expression of MITF.

It has been reported that several signaling pathways are involved in regulating melanin synthesis. The extracellular signal-regulated kinase (ERK) signaling pathway induces the inhibition of melanin synthesis in mouse B16 melanoma cells [6]. The activation of ERK leads to phosphorylation of MITF at serine 73, which results in MITF ubiquitination and degradation [7-9]. Additionally, LY294002, a specific inhibitor of the Akt pathway, triggers melanogenesis in B16 cells [10]. Thus, the activation of Akt is implicated in modulating melanogenesis [11].

Sphingolipids are known to function as key signaling messengers in a variety of cellular processes such as cell growth, differentiation, cell death, and cell movement [12,13]. In recent years, many reports have shown that sphingolipids are deeply involved in regulating melanin synthesis. It has been reported that the sphingolipid metabolites ceramide and sphingosine-1-phosphate inhibit melanogenesis in melanocytes [9,14-16]. Sphingosylphosphorylcholine (SPC), another sphingolipid, is produced by the N-deacylation of sphingomyelin and has been reported to act as a signaling molecule in various biologic processes [17,18]. It was found that SPC stimulates melanin synthesis in human melanocytes [19]. On the other hand, we reported that SPC reduces melanogenesis via ERK activation in human and mouse melanocytes [20,21]. To understand these conflicting results, the molecular mechanisms of SPC responsible for melanogenesis should be completely elucidated. In the present study, we further examined the effects of SPC on melanogenesis and SPC-modulated signaling pathways in Mel-Ab cells.

## Materials and methods

### Reagents

SPC was purchased from Avanti Polar Lipids (Alabaster, AL, USA); LY294002 and rapamycin were from Cell Signaling Technology (Beverly, MA, USA). Fetal bovine serum (FBS) was obtained from Hyclone (Logan, UT, USA), and Complete™ protease inhibitor cocktail was from Roche (Mannheim, Germany). Cholera toxin (CT), 12-O-tetradecanoylphorbol-13-acetate (TPA), Triton X-100, Tris, β-mercaptoethanol, phenylmethylsulfonyl fluoride, fatty acid-free bovine serum albumin (BSA), synthetic melanin, α-MSH, and L-DOPA were all purchased from Sigma (St. Louis, MO, USA). Antibodies recognizing phosphorylated Akt (Ser473, no. 9271), total Akt (no. 4691), phosphorylated mTOR (no. 2971), and total mTOR (no. 2972) were obtained from Cell Signaling Technology. Microphthalmia Ab-1 (C5, MS-771-P0) was from NeoMarkers (Fremont, CA, USA), and anti-actin (I-19) antibody was purchased from Santa Cruz Biotechnology, Inc. (Santa Cruz, CA, USA).

### Cell cultures

The Mel-Ab cell line is a mouse-derived spontaneously immortalized melanocyte cell line that synthesizes large quantities of melanin [22]. Mel-Ab cells were maintained in DMEM supplemented with 10% FBS, 100 nM TPA, 1 nM CT, 50 μg/mL streptomycin, and 50 U/mL penicillin at 37°C in 5% CO_2_. B16/F10 murine melanoma cells were cultured in DMEM supplemented with 10% FBS, 50 μg/mL streptomycin, and 50 U/mL penicillin at 37°C in 5% CO_2_.

### Cell viability assay

Cell viability was measured using a crystal violet assay. After incubation with SPC for 24 h, the culture media was removed. Mel-Ab cells were stained with 0.1% crystal violet in 10% ethanol for 5 min at room temperature then rinsed 4 times with distilled water. The crystal violet retained by adherent cells was extracted with 95% ethanol, and the absorbance was determined at 590 nm using an ELISA reader (VERSAMax; Molecular Devices, Sunnyvale, CA, USA).

### Assessment of melanin contents and microscopy

Mel-Ab cells were incubated with SPC for 4 d, and were observed under a phase contrast microscope (Olympus Optical Co., Tokyo, Japan) and photographed using a DCM300 digital camera (Scopetek, Inc., Hangzhou, China) supported by ScopePhoto software (Scopetek, Inc.). The melanin contents of the cells were analyzed as previously described [23] with some modifications. Cell pellets were dissolved in 1 mL of 1 N NaOH at 100°C for 30 min and centrifuged for 20 min at 16,000 × g. The optical densities (OD) of the supernatants were assessed at 400 nm using an ELISA reader. Standard curves were prepared with synthetic melanin (0 - 300 μg/mL) in triplicate for each experiment.

### Tyrosinase activity

Tyrosinase activity was analyzed using the method described by Busca *et al*. [10] with slight modification. In brief, Mel-Ab cells were seeded in 6-well plates and incubated with SPC for 4 d. The cells were washed with ice-cold PBS, lysed with phosphate buffer (pH 6.8) containing 1% Triton X-100, and disrupted by freezing and thawing. After quantifying the protein levels of the lysate and adjusting the protein concentrations with lysis buffer, 90 μL of each lysate containing the same amount of protein was placed in each well of a 96-well plate, and 10 μL of 10 mM L-DOPA was then added to each well. The control wells contained 90 μL of lysis buffer and 10 μL of 10 mM L-DOPA. Following incubation at 37°C for 20 min, the absorbance of each well was measured at 475 nm using an ELISA reader.

### Western blot analysis

Mel-Ab cells were lysed in cell lysis buffer containing 62.5 mM Tris-HCl (pH 6.8), 2% SDS, 5% β-mercaptoethanol, 2 mM phenylmethylsulfonyl fluoride, protease inhibitor cocktail, 1 mM Na_3_VO_4_, 50 mM NaF, and 10 mM EDTA. Proteins were separated by SDS-polyacrylamide gel electrophoresis and blotted onto PVDF membranes, which were then blocked with 5% skim milk in Tris-buffered saline containing 0.05% Tween 20. The blots were incubated with the appropriate primary antibodies at a dilution of 1:1000, and then further incubated with horseradish peroxidase-conjugated secondary antibody. The blots were developed by a chemiluminescent substrate (Pierce, Rockford, IL, USA). The images of the membranes were obtained using a LAS-1000 lumino-image analyzer (Fuji Film, Tokyo, Japan).

### Transfection and luciferase assay

B16/F10 melanoma cells were cultured in 60 mm dishes and transfected using the GenePORTER transfection reagent according to the manufacturer's recommendations (Gene Therapy Systems, San Diego, CA, USA). The luciferase reporter plasmid (pMITF) which contains the fragment of the mouse MITF promoter (pMI; -2135/+136) in pGL_2_B vector was kindly provided by Dr. R. Ballotti (Nice, France) [24]. To examine the effects of SPC, cells were transfected with 2 μg per well of the reporter plasmid and 1 μg of pSV-β-galactosidase vector (Promega, Madison, WI, USA) as a control for transfection efficiency variability. After transfection, cells were treated with SPC for 24 h in the absence or presence of α-MSH and then, the cells were processed using a Luciferase Assay Kit (Applied Biosystems, Bedford, MA, USA). Soluble extracts were analyzed for luciferase and β-galactosidase activities.

### Statistical analysis

The statistical significance of the differences between groups was assessed by analysis of variance (ANOVA), followed by the Student's *t*-test. *P *values < 0.01 were considered significant.

## Results

### Effect of SPC on Mel-Ab cell viability

**Figure 1:**
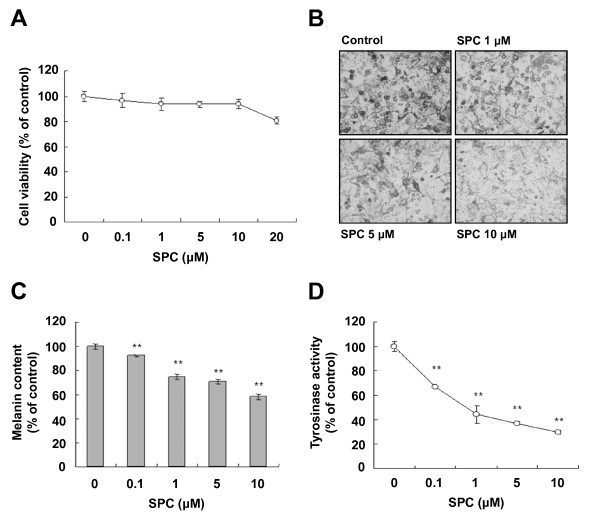
**Effects of SPC on melanogenesis in Mel-Ab cells**. (A) Cells were treated with SPC at various concentrations (0-20 μM) for 24 h and cell viability was determined using a crystal violet assay. (B) Cells were incubated with 0-10 μM SPC for 4 d, and phase contrast microscopy photographs were obtained using a digital video camera. Melanin contents (C) and tyrosine activity (D) were analyzed as described in 'Materials and Methods'. Data represent the mean ± SD of triplicate assays expressed as percentages of the control. **P < 0.01 compared to the untreated control.

The effect of SPC on Mel-Ab cell viability was determined using a crystal violet assay. Mel-Ab cells were treated with SPC at concentrations of 0.1-20 μM. Treatment of SPC exhibited no effects on the viability of Mel-Ab cells over a concentration range of 0.1-10 μM, indicating that SPC was not cytotoxic to Mel-Ab cells at a concentration of 0.1-10 μM (Figure 1A).

### Effects of SPC on melanin synthesis and tyrosinase activity in Mel-Ab cells

We previously reported that SPC suppresses melanin production in normal human melanocytes [20]. To examine the effect of SPC on melanogenesis in Mel-Ab cells, cells were treated with SPC at concentrations of 0.1-10 μM. Following SPC treatment for 4 d, the cells were observed under a phase contrast microscope. As shown in Figure 1B, SPC-treated Mel-Ab cells showed a reduction in melanin pigmentation in a dose-dependent manner. Moreover, SPC treatment significantly reduced the melanin content of Mel-Ab cells (Figure 1C), indicating that SPC induces significant hypopigmentation. In addition, we examined tyrosinase activity in Mel-Ab cells exposed to SPC and observed that SPC significantly inhibited tyrosinase activity in a concentration-dependent manner (Figure 1D).

### SPC reduces MITF transcription and protein levels

**Figure 2:**
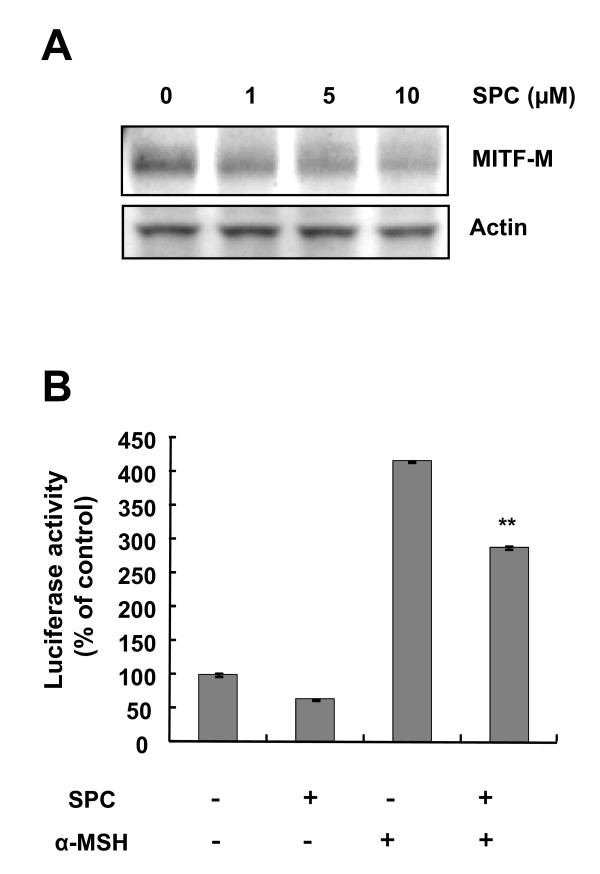
**SPC induces MITF downregulation in Mel-Ab cells**. (A) After serum starvation for 24 h, Mel-Ab cells were treated with 0-10 μM SPC for 3 h. Whole cell lysates were analyzed by Western blotting with antibodies against MITF-M and actin (loading control). (B) B16/F10 cells were transfected with 2 μg of luciferase reporter plasmid plus 1 μg of the pSV-β-galactosidase control vector. After incubating with 10 μM SPC for 24 h, luciferase activity was assessed and normalized with respect to β-galactosidase activity. Results are expressed as percentages of the untreated control. Each determination was made in triplicate; the data shown represent means ± SD. **P <0.01 compared to the α-MSH-treated cells.

Next, we examined whether SPC induces MITF downregulation in Mel-Ab cells. As shown in Figure 2A, SPC decreased melanocyte specific MITF (MITF-M) protein levels in a dose-dependent manner in Mel-Ab cells. We further investigated whether SPC regulates the expression of MITF by reducing transcription activity of the MITF promoter. Treatment with SPC suppressed MITF promoter activity induced by α-MSH in B16 melanoma cells, indicating that SPC blocks the transcription of MITF (Figure 2B).

### The mTOR signaling pathway is involved in SPC-induced hypopigmentation

**Figure 3:**
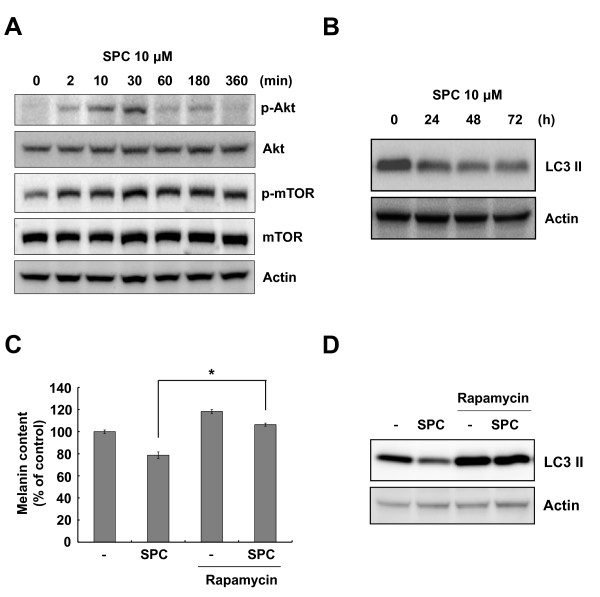
**SPC activates Akt and mTOR in Mel-Ab cells**. (A) After serum starvation for 24 h, Mel-Ab cells were treated with 10 μM SPC for the indicated times. Whole cell lysates were analyzed by Western blotting with antibodies against phospho-Akt, Akt, phospho-mTOR, mTOR, and actin (loading control). (B) Cells were incubated with 10 μM SPC for the indicated time periods and lysed. The levels of LC3 II were analyzed by Western blotting. (C) Cells were pretreated with 100 nM rapamycin for 1 h prior to the addition of 10 μM SPC. Cells were incubated for an additional 4 d and then the cellular melanin contents were analyzed. Data are expressed as the mean ± SD of triplicate assays. *P <0.05 compared to the SPC-treated cells. (D) Cells were preincubated with 100 nM rapamycin for 1 h prior to addition of 10 μM SPC and then incubated for another 3 d. Whole cell lysates were analyzed by Western blotting with antibodies against LC3 II and actin (loading control).

**Figure 4:**
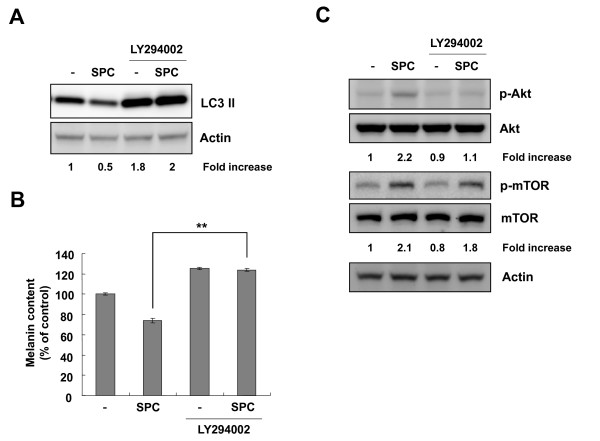
**SPC stimulates mTOR via the Akt signaling pathway in Mel-Ab cells**. Cells were preincubated with 20 μM LY294002 for 30 min prior to the addition of 10 μM SPC and then incubated for another 3 d. (A) Cells were lysed and the levels of LC3 II were analyzed by Western blotting. Actin was used as a loading control. (B) Melanin content was measured as described in the Materials and Methods. Data represent means ± SD of triplicate experiments. ** *P <*0.01 compared to the SPC-treated cells. (C) After serum starvation for 24 h, cells were incubated with 10 μM SPC for 30 min in the presence or absence of 20 μM LY294002. Whole cell lysates were analyzed by Western blotting with antibodies against phospho-Akt, Akt, phospho-mTOR, mTOR, and actin (loading control). Fold increases over the control were determined by densitometric analysis and are shown below each lane.

We recently reported that SPC-induced Akt activation blocks melanin synthesis in Mel-Ab cells [21]. Recent studies have also demonstrated that Akt activates serine/threonine mTOR protein kinase [25]. Therefore, we investigated whether SPC induces activation of mTOR. As shown in Figure 3A, SPC induced not only Akt phosphorylation but also mTOR phosphorylation. Because mTOR is known to regulate the accumulation of LC3 II [26], we examined the level of LC3 II after SPC treatment. SPC-treated cells showed a continuous reduction of LC3 II levels (Figure 3B). Because SPC triggered the activation of mTOR (Figure 3A), cells were incubated with SPC in the presence or absence of rapamycin, a specific mTOR inhibitor. Addition of rapamycin significantly abolished the inhibition of melanin synthesis in SPC-treated cells (Figure 3C). Moreover, rapamycin restored the production of LC3 II downregulated by SPC (Figure 3D), indicating that SPC-induced activation of mTOR was inhibited by rapamycin. Because Akt is known to activate mTOR, cells were treated with SPC in the presence or absence of LY294002, a specific Akt pathway inhibitor. As shown in Figure 4A, addition of LY294002 abrogated the reduction of LC3 II levels in SPC-treated cells. It was also found that LY294002 eliminated melanin synthesis inhibition by SPC (Figure 4B). Moreover, LY294002 inhibited the Akt phosphorylation and suppressed mTOR phosphorylation in SPC-treated cells (Figure 4C). These results indicate that the Akt and mTOR signaling pathways may be involved in SPC-modulated melanin synthesis.

## Discussion

Sphingolipids have been reported to regulate melanin synthesis in mouse and human melanocytes [9,14,15]. In the present study, we confirmed that SPC inhibits melanin synthesis in mouse melanocytes. Moreover, our results revealed that SPC treatment for 24 h suppressed the transcription activity of MITF. However, we previously reported that there is no change in the level of MITF mRNA in Mel-Ab cells until 6 h after SPC treatment, but MITF protein levels are reduced 24 h post-SPC treatment [21]. Because the proteasome inhibitor MG132 mostly prevents SPC-induced MITF expression downregulation, we concluded that the reduced MITF levels were due to MITF degradation. As mentioned, however, the transcription activity of MITF was reduced by long-term SPC treatment. Thus, SPC-induced MITF downregulation may result from both inhibition of MITF transcription activity and MITF degradation.

It has been suggested that the Akt signaling pathway is related to regulation of melanin synthesis [10,11]. It was reported that glycogen synthase kinase 3β (GSK3β) phosphorylates MITF at serine 298, consequently augmenting the binding of MITF to the tyrosinase promoter [27]. In addition, GSK3β is known to be phosphorylated and inactivated by Akt [28]. In a previous report, we have shown that Akt activation triggered by SPC regulates melanogenesis via G-protein-coupled receptors [21]. In the present study, we investigated whether there are any other downstream effectors of the Akt signaling pathway in SPC-treated melanocytes.

One of the important downstream targets of Akt is mTOR, which controls cell growth and proliferation [29]. Recently, it has been reported that activation of the serine/threonine mTOR protein kinase is involved in the inhibition of melanin synthesis in B16 melanoma cells [30]. Because SPC triggers the activation of Akt, we examined whether SPC activates mTOR. As expected, treatment with SPC induced the activation of mTOR in Mel-Ab cells (Figure 3A). Since mTOR is known to be a critical signaling factor which inhibits the accumulation of LC3 II, an autophagosomal marker, we examined the effect of SPC on LC3 II levels. SPC-treated cells showed a decrease of LC3 II, indicating that the activation of mTOR by SPC may regulate the level of LC3 II (Figure 3B). Treatment with rapamycin, a specific mTOR pathway inhibitor, reversed the inhibition of melanin synthesis and decrease of LC3 II level by SPC. Although LY294002, a specific inhibitor of the Akt pathway, partially inhibited mTOR phosphorylation in SPC-treated Mel-Ab cells, it completely restored a decrease of LC3 II by SPC. These results indicate that activation of mTOR may be partially due to the activation of Akt by SPC, and mTOR may be regulated through another pathway in SPC-treated Mel-Ab cells. In previous studies, it has been reported that mTOR was activated by phospholipase D1 (PLD1), which plays a negative regulatory role in melanogenesis [30], and that WIPI1 depletion stimulated the activation of mTOR, leading to inhibition of melanosome maturation [31]. Thus, additional investigation is needed to clarify whether there is another upstream regulator of mTOR in SPC-treated melanocytes. Interestingly, it was reported that autophagic and melanosomal markers co-localize in mature melanosomes, indicating that autophagy-related factors may be involved in melanogenesis [32]. These findings raise the possibility that a relationship may exist between autophagy and melanogenesis. Further investigations are currently underway to elucidate this possibility.

## Conclusions

In summary, the present study demonstrated that SPC has hypopigmentation effects by regulating both the mRNA and protein levels of MITF, a key transcription regulator in melanogenesis. Moreover, our data suggest that the mTOR signaling pathway may participate in the SPC regulation of melanin synthesis.

## Abbreviations

BSA: bovine serum albumin; CT: cholera toxin; ERK: extracellular signal-regulated kinase; FBS: fetal bovine serum; GSK3β: glycogen synthase kinase 3β; MITF: microphthalmia-associated transcription factor; mTOR: mammalian target of rapamycin; OD: optical density; SPC: sphingosylphosphorylcholine; TPA: 12-O-tetradecanoylphorbol-13-acetate; TRP: tyrosinase-related protein.

## Competing interests

The authors declare that they have no competing interests.

## Authors' contributions

HSJ participated in data acquisition, interpretation, and the writing of this manuscript. SHL, HYY, KJB, NSK, and KCP participated in the study design and data interpretation. DSK contributed to the experimental design, data interpretation, editing, and submission of this manuscript. All authors read and approved the final manuscript.

## References

[B1] Costin GE Hearing VJ Human skin pigmentation: melanocytes modulate skin color in response to stress FASEB J 2007 21 976 994 10.1096/fj.06-6649rev 17242160

[B2] Ando H Kondoh H Ichihashi M Hearing VJ Approaches to identify inhibitors of melanin biosynthesis via the quality control of tyrosinase J Invest Dermatol 2007 127 751 761 10.1038/sj.jid.5700683 17218941

[B3] Schallreuter KU Hasse S Rokos H Chavan B Shalbaf M Spencer JD Wood JM Cholesterol regulates melanogenesis in human epidermal melanocytes and melanoma cells Exp Dermatol 2009 18 680 688 10.1111/j.1600-0625.2009.00850.x 19469904

[B4] Levy C Khaled M Fisher DE MITF: master regulator of melanocyte development and melanoma oncogene Trends Mol Med 2006 12 406 414 10.1016/j.molmed.2006.07.008 16899407

[B5] Villareal MO Han J Yamada P Shigemori H Isoda H Hirseins inhibit melanogenesis by regulating the gene expressions of Mitf and melanogenesis enzymes Exp Dermatol 2010 19 450 457 10.1111/j.1600-0625.2009.00964.x 19765058

[B6] Englaro W Bertolotto C Busca R Brunet A Pages G Ortonne JP Ballotti R Inhibition of the mitogen-activated protein kinase pathway triggers B16 melanoma cell differentiation J Biol Chem 1998 273 9966 9970 10.1074/jbc.273.16.9966 9545341

[B7] Wu M Hemesath TJ Takemoto CM Horstmann MA Wells AG Price ER Fisher DZ Fisher DE c-Kit triggers dual phosphorylations, which couple activation and degradation of the essential melanocyte factor Mi Genes Dev 2000 14 301 312 10673502 PMC316361

[B8] Xu W Gong L Haddad MM Bischof O Campisi J Yeh ET Medrano EE Regulation of microphthalmia-associated transcription factor MITF protein levels by association with the ubiquitin-conjugating enzyme hUBC9 Exp Cell Res 2000 255 135 143 10.1006/excr.2000.4803 10694430

[B9] Kim DS Hwang ES Lee JE Kim SY Kwon SB Park KC Sphingosine-1-phosphate decreases melanin synthesis via sustained ERK activation and subsequent MITF degradation J Cell Sci 2003 116 1699 1706 10.1242/jcs.00366 12665551

[B10] Busca R Bertolotto C Ortonne JP Ballotti R Inhibition of the phosphatidylinositol 3-kinase/p70(S6)-kinase pathway induces B16 melanoma cell differentiation J Biol Chem 1996 271 31824 31830 10.1074/jbc.271.50.31824 8943224

[B11] Oka M Nagai H Ando H Fukunaga M Matsumura M Araki K Ogawa W Miki T Sakaue M Tsukamoto K Konishi H Kikkawa U Ichihashi M Regulation of melanogenesis through phosphatidylinositol 3-kinase-Akt pathway in human G361 melanoma cells J Invest Dermatol 2000 115 699 703 10.1046/j.1523-1747.2000.00095.x 10998146

[B12] Dobrowsky RT Sphingolipid signalling domains floating on rafts or buried in caves? Cell Signal 2000 12 81 90 10.1016/S0898-6568(99)00072-8 10679576

[B13] Spiegel S Milstien S Sphingosine-1-phosphate: an enigmatic signalling lipid Nat Rev Mol Cell Biol 2003 4 397 407 10.1038/nrm1103 12728273

[B14] Kim DS Kim SY Moon SJ Chung JH Kim KH Cho KH Park KC Ceramide inhibits cell proliferation through Akt/PKB inactivation and decreases melanin synthesis in Mel-Ab cells Pigment Cell Res 2001 14 110 115 10.1034/j.1600-0749.2001.140206.x 11310790

[B15] Kim DS Kim SY Chung JH Kim KH Eun HC Park KC Delayed ERK activation by ceramide reduces melanin synthesis in human melanocytes Cell Signal 2002 14 779 785 10.1016/S0898-6568(02)00024-4 12034359

[B16] Kim DS Park SH Kwon SB Youn SW Park KC Effects of lysophosphatidic acid on melanogenesis Chem Phys Lipids 2004 127 199 206 10.1016/j.chemphyslip.2003.11.002 14726002

[B17] Desai NN Spiegel S Sphingosylphosphorylcholine is a remarkably potent mitogen for a variety of cell lines Biochem Biophys Res Commun 1991 181 361 366 10.1016/S0006-291X(05)81427-5 1958205

[B18] Desai NN Carlson RO Mattie ME Olivera A Buckley NE Seki T Brooker G Spiegel S Signaling pathways for sphingosylphosphorylcholine-mediated mitogenesis in Swiss 3T3 fibroblasts J Cell Biol 1993 121 1385 1395 10.1083/jcb.121.6.1385 8389770 PMC2119705

[B19] Higuchi K Kawashima M Ichikawa Y Imokawa G Sphingosylphosphorylcholine is a Melanogenic Stimulator for Human Melanocytes Pigment Cell Res 2003 16 670 678 10.1046/j.1600-0749.2003.00097.x 14629725

[B20] Kim DS Park SH Kwon SB Park ES Huh CH Youn SW Park KC Sphingosylphosphorylcholine-induced ERK activation inhibits melanin synthesis in human melanocytes Pigment Cell Res 2006 19 146 153 10.1111/j.1600-0749.2005.00287.x 16524430

[B21] Kim DS Park SH Kwon SB Kwon NS Park KC Sphingosylphosphorylcholine inhibits melanin synthesis via pertussis toxin-sensitive MITF degradation J Pharm Pharmacol 2010 62 181 187 20487197 10.1211/jpp.62.02.0005

[B22] Dooley TP Gadwood RC Kilgore K Thomasco LM Development of an in vitro primary screen for skin depigmentation and antimelanoma agents Skin Pharmacol 1994 7 188 200 10.1159/000211294 8024800

[B23] Tsuboi T Kondoh H Hiratsuka J Mishima Y Enhanced melanogenesis induced by tyrosinase gene-transfer increases boron-uptake and killing effect of boron neutron capture therapy for amelanotic melanoma Pigment Cell Res 1998 11 275 282 10.1111/j.1600-0749.1998.tb00736.x 9877098

[B24] Khaled M Larribere L Bille K Aberdam E Ortonne JP Ballotti R Bertolotto C Glycogen synthase kinase 3beta is activated by cAMP and plays an active role in the regulation of melanogenesis J Biol Chem 2002 277 33690 33697 10.1074/jbc.M202939200 12093801

[B25] Shaw RJ Cantley LC Ras, PI(3)K and mTOR signalling controls tumour cell growth Nature 2006 441 424 430 10.1038/nature04869 16724053

[B26] Spilman P Podlutskaya N Hart MJ Debnath J Gorostiza O Bredesen D Richardson A Strong R Galvan V Inhibition of mTOR by rapamycin abolishes cognitive deficits and reduces amyloid-beta levels in a mouse model of Alzheimer's disease PLoS One 2010 5 e9979 10.1371/journal.pone.0009979 20376313 PMC2848616

[B27] Takeda K Takemoto C Kobayashi I Watanabe A Nobukuni Y Fisher DE Tachibana M Ser298 of MITF, a mutation site in Waardenburg syndrome type 2, is a phosphorylation site with functional significance Hum Mol Genet 2000 9 125 132 10.1093/hmg/9.1.125 10587587

[B28] Cross DA Alessi DR Cohen P Andjelkovich M Hemmings BA Inhibition of glycogen synthase kinase-3 by insulin mediated by protein kinase B Nature 1995 378 785 789 10.1038/378785a0 8524413

[B29] Wullschleger S Loewith R Hall MN TOR signaling in growth and metabolism Cell 2006 124 471 484 10.1016/j.cell.2006.01.016 16469695

[B30] Ohguchi K Banno Y Nakagawa Y Akao Y Nozawa Y Negative regulation of melanogenesis by phospholipase D1 through mTOR/p70 S6 kinase 1 signaling in mouse B16 melanoma cells J Cell Physiol 2005 205 444 451 10.1002/jcp.20421 15895362

[B31] Ho H Kapadia R Al-Tahan S Ahmad S Ganesan AK WIPI1 coordinates melanogenic gene transcription and melanosome formation via TORC1 inhibition J Biol Chem 2011 286 12509 12523 10.1074/jbc.M110.200543 21317285 PMC3069453

[B32] Ganesan AK Ho H Bodemann B Petersen S Aruri J Koshy S Richardson Z Le LQ Krasieva T Roth MG Farmer P White MA Genome-wide siRNA-based functional genomics of pigmentation identifies novel genes and pathways that impact melanogenesis in human cells PLoS Genet 2008 4 e1000298 10.1371/journal.pgen.1000298 19057677 PMC2585813

